# Visual Blood, Visualisation of Blood Gas Analysis in Virtual Reality, Leads to More Correct Diagnoses: A Computer-Based, Multicentre, Simulation Study

**DOI:** 10.3390/bioengineering10030340

**Published:** 2023-03-08

**Authors:** Lisa Bergauer, Samira Akbas, Julia Braun, Michael T. Ganter, Patrick Meybohm, Sebastian Hottenrott, Kai Zacharowski, Florian J. Raimann, Eva Rivas, Manuel López-Baamonde, Donat R. Spahn, Christoph B. Noethiger, David W. Tscholl, Tadzio R. Roche

**Affiliations:** 1 Institute of Anaesthesiology, University Hospital Zurich, 8091 Zurich, Switzerland; 2 Departments of Epidemiology and Biostatistics, Epidemiology, Biostatistics and Prevention Institute, University of Zurich, 8001 Zurich, Switzerland; 3 Institute of Anaesthesiology and Intensive Care Medicine, Clinic Hirslanden Zurich, 8032 Zurich, Switzerland; 4 Department of Anaesthesiology, Intensive Care, Emergency, and Pain Medicine, University Hospital Wuerzburg, University of Wuerzburg, 97080 Wuerzburg, Germany; 5 Department of Anaesthesiology, Intensive Care Medicine, and Pain Therapy, University Hospital Frankfurt, Goethe University Frankfurt, 60323 Frankfurt, Germany; 6 Department of Anaesthesiology, Intensive Care Medicine, and Pain Therapy, Hospital Clinic of Barcelona, University of Barcelona, 08036 Barcelona, Spain

**Keywords:** virtual reality, blood gas analysis, data display, point-of-care, situation awareness, user-centred design, diagnostic correctness

## Abstract

Interpreting blood gas analysis results can be challenging for the clinician, especially in stressful situations under time pressure. To foster fast and correct interpretation of blood gas results, we developed Visual Blood. This computer-based, multicentre, noninferiority study compared Visual Blood and conventional arterial blood gas (ABG) printouts. We presented six scenarios to anaesthesiologists, once with Visual Blood and once with the conventional ABG printout. The primary outcome was ABG parameter perception. The secondary outcomes included correct clinical diagnoses, perceived diagnostic confidence, and perceived workload. To analyse the results, we used mixed models and matched odds ratios. Analysing 300 within-subject cases, we showed noninferiority of Visual Blood compared to ABG printouts concerning the rate of correctly perceived ABG parameters (rate ratio, 0.96; 95% CI, 0.92–1.00; *p* = 0.06). Additionally, the study revealed two times higher odds of making the correct clinical diagnosis using Visual Blood (OR, 2.16; 95% CI, 1.42–3.29; *p* < 0.001) than using ABG printouts. There was no or, respectively, weak evidence for a difference in diagnostic confidence (OR, 0.84; 95% CI, 0.58–1.21; *p* = 0.34) and perceived workload (Coefficient, 2.44; 95% CI, −0.09–4.98; *p* = 0.06). This study showed that participants did not perceive the ABG parameters better, but using Visual Blood resulted in more correct clinical diagnoses than using conventional ABG printouts. This suggests that Visual Blood allows for a higher level of situation awareness beyond individual parameters’ perception. However, the study also highlighted the limitations of today’s virtual reality headsets and Visual Blood.

## 1. Introduction

Since the introduction of blood gas analysis into clinical practice in the 1960s, biochemical and technological research have progressed rapidly [1,2], resulting in blood gas analysis becoming one of today’s most common point-of-care tests (POCT) [3]. This POC method provides valuable information about the patient’s acid–base, gas exchange, metabolic, and electrolyte status in a matter of seconds [4]. The accurate and timely interpretation of results is essential to identify and treat pathophysiological processes [3,5]. According to the three-stage concept of situation awareness by Mica Endsley, the perception of the elements, in this case individual blood values, is initially necessary. Hereon builds the second level of comprehension, meaning the correct interpretation of the information obtained [6]. Errors related to the different levels of situation awareness are considered a leading cause of mistakes in medicine, especially in stressful and dynamic situations [7,8]. In critically ill or unstable patients, many individual blood gas values differ from the norm. Standard patient data management systems (PDMS) or paper printouts only mark the pathologies with arrows or colour changes in a long numerical list. However, such presentations quickly become confusing in the case of numerous changes and fail to draw attention due to an overload of information [5]. There is ample evidence that supports the importance of design characteristics on situation awareness and decision-making for cognitive aids and patient monitoring in medicine [9,10,11,12]. Information systems should be designed according to user-centred design principles to foster the cognitive process of perception and comprehension [7].

In recent years, various visualisation technologies have been developed to display medical data, including Hamilton’s dynamic lung [13,14]. In a simulation study, the graphical representation of the patient monitor helped clinicians to detect and better manage critical cardiovascular events more quickly [15,16]. Furthermore, new display techniques in immersive virtual environments, including virtual reality (VR) and augmented reality (AR), have an untapped potential to foster information transfer and reduce the users’ cognitive load [17,18,19]. So far, scant attention has been paid to improve the interface between users and the display of blood gas analysis results. The idea of Visual Blood builds on this approach and intends to assist health care professionals in interpreting blood gas analysis results. We developed the new visualisation technique, Visual Blood, as a three-dimensional virtual reality animation that illustrates the measured values and is optimised by considering user-centred design principles and aspiring for better situation awareness. This study compares Visual Blood with conventional arterial blood gas (ABG) printouts in a noninferiority trial in terms of the correct perception of individual blood gas parameters. In addition, we examined correctly assigned clinical diagnoses, perceived diagnostic confidence, and perceived workload.

## 2. Materials and Methods

Before starting this project, we submitted the study protocol to the responsible local ethics committees in Zurich, Frankfurt, Wuerzburg, and Barcelona, which issued declarations of no objection. Furthermore, all participants agreed in written form with the recording, statistical analysis, and publication of the anonymised data. We report the methods and results of this study in accordance with the extension of the CONSORT 2010 statement “Reporting of noninferiority and equivalence randomized trials” [20].

### 2.1. Visual Blood

**Figure 1:**
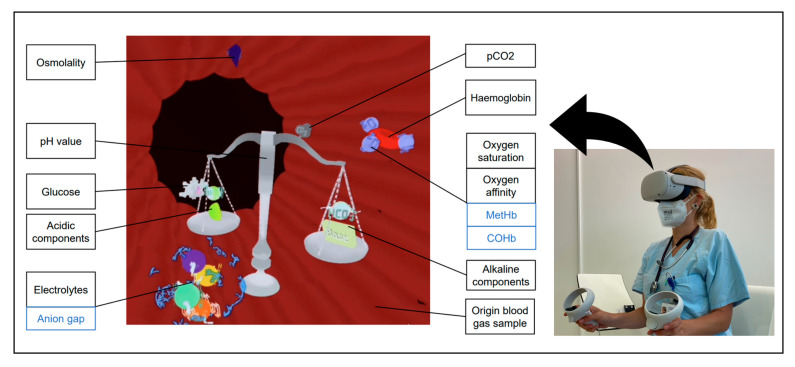
Normal blood gas sample in Visual Blood. The red vessel wall indicates an arterial sample. The pH value is shown in the form of a scale. In the case of an imbalance, this can be specified by the appearance of a number of acidic or alkaline icons. Acidic components include lactate or a high number of CO_2_ clouds accumulating around the respective side of the scale. The alkaline section of the scale contains bicarbonate and base excess. Visual Blood displays the electrolytes sodium, potassium, chloride, and calcium in a colour code. The values written in blue are only displayed in the case of a deviation from the reference range, accordingly not visible in this example. In the case of oxygen partial pressure that is too high, a large light blue cloud appears. COHb, carboxyhaemoglobin. MetHb, methaemoglobin. pCO_2_, carbon dioxide partial pressure.

We created a three-dimensional, animated visualisation of the values displayed by default in ABG samples, integrating user-centred design characteristics, such as simplification, intuitiveness, and parallel information transfer [7,21]. Visual Blood was optimised for the VR headset Quest 2 (Oculus, Meta Platforms, Inc., Menlo Park, CA, USA). However, the technique also works with other VR headsets or on a conventional monitor. In the visualisation, the user is situated inside the blood vessel and perceives the components and their interactions not as numerical values but as intuitive icons. For example, Visual Blood depicts the acid–base balance in the form of a scale, whose one side is loaded with acidic components and the other with alkaline components. In the case of a measured abnormal pH value, the amount of acidic or basic valences on the scale increases or decreases, and the balance of the scale shifts accordingly (Figure 1).

Visual Blood displays 18 different blood gas parameters (Figure 1). In the “Visual Blood Educational Experience” (Video S1 in Supplementary Materials), we explain all the parameters and their deviations. For further illustration, Supplementary Figure S1 shows exemplary scenario 2, “acute respiratory acidosis,” as visible blood and two conventional presentations used at different study sites.

### 2.2. Study Design and Participants

This was an investigator-initiated, prospective, randomised, computer-based, multicentre, multinational within-subject simulation study that compared Visual Blood and conventional ABG printouts. We included participants from five tertiary care hospitals: The University Hospital Zurich and Hirslanden Clinic of Zurich in Switzerland, the University Hospitals Frankfurt and Wuerzburg in Germany, and the Hospital Clinic de Barcelona in Spain. We recruited ten anaesthesiologists who were either doing their residency or already board certified at each centre, adding up to 50 subjects. Participation was voluntary without financial compensation and based on availability from clinical work.

### 2.3. Study Procedure

We welcomed the participants and asked them to answer a brief demographic survey. Subsequently, we presented the teaching video “Visual Blood Educational Experience” (Video S1 in Supplementary Materials) via VR headset, which enabled the participants to acquaint themselves with the new technique. In addition, the participants surveyed an example of the conventional ABG, which corresponded in layout to the centre’s respective ABG printouts.

We then showed six different scenarios of ABG results related to clinical diagnoses, either as Visual Blood or as a matching conventional ABG printout, resulting in twelve cases per participant. Each participant received all twelve cases in randomised order (ResearchRandomizer v4.0; http://www.randomizer.org; accessed on 15 May 2021). We showed each scenario as Visual Blood via VR headset or as an ABG printout for exactly 15 s. The participants’ task was to memorise the status of the parameters displayed and appraise a clinical diagnosis. We requested the participants to rate all blood gas parameters presented, whether they were too low, safe, or too high.

Furthermore, the participants matched the ABG results observed to a clinical diagnosis. After each case, the participants evaluated their diagnostic confidence and perceived workload in fulfilling the given tasks. Finally, to complete the study, the participants evaluated four general statements on a 5-point Likert scale (from strongly disagree to strongly agree) to collect subjective impressions regarding Visual Blood and VR in clinical practice. All answers were entered in a programmed survey (Harvest your data, Wellington, New Zealand) on an iPad (Apple Inc., Cupertino, CA, USA). We used Adobe Acrobat Pro 2020 (Adobe Inc., San José, CA, USA) to adopt all ABG printouts to format templates with local reference values and units to ensure a high level of familiarity in every centre. Table S1 in Supplementary Materials provides an overview of all scenarios with the respective normal and deranged parameters.

### 2.4. Outcome Measures

The study’s primary outcome was ABG parameter perception, defined as the number of correctly perceived blood gas parameters per case. The participants had to indicate whether each parameter (18 per case) was too low, safe, or too high.

As a secondary outcome, we investigated the clinical diagnoses (binary as correct/incorrect), whereby participants could choose from twelve answers of which one matched the correct diagnosis for the scenario.

Further secondary outcomes included the perceived diagnostic confidence and the perceived workload. The participants rated their subjective diagnostic confidence (binary as unconfident/confident) and perceived workload using the National Aeronautics and Space Administration Task Load Index (NASA TLX; from 0 to 100). The latter is a validated questionnaire containing the arithmetic mean of six sub-questions [22,23,24]. We dropped one of the six questions because our study had no physical effort.

### 2.5. Statistical Analysis

For the descriptive statistics, we show medians and interquartile ranges (IQR: 25/75 percentile) for the continuous data and numbers and percentages for the categorical data.

For the noninferiority analysis of the primary outcome, the number of correctly perceived ABG parameters per case, we used a mixed Poisson regression model with a random intercept per participant. As there is no established noninferiority margin for visualisation comparisons of ABG results, we defined a proprietary margin for this study based on clinical experience and literature from other visualization projects [16]. We set the noninferiority margin at 10%, corresponding to a rate ratio (RR) of 0.9. We considered noninferiority as established if the lower bound of the 95% confidence interval (95% CI) was above that margin.

For evaluating the perception of each ABG parameter individually (binary as correct or incorrect perception), we computed crude matched odds ratios (OR) per parameter as an addition to the overall assessment with the mixed Poisson regression model mentioned. This enabled us to determine the ABG parameter presentations that performed particularly well or particularly poorly in Visual Blood.

Mixed logistic regression models with a random intercept per participant were used to analyse repeated binary data (secondary outcome—correct clinical diagnoses, and secondary outcome—perceived diagnostic confidence). To analyse the overall NASA TLX that represented the perceived workload as a continuous outcome, we ran a linear mixed model that included a random intercept for each participant. We adjusted all models for potentially relevant covariates, such as age, gender, work experience, study centre, scenario, and self-rated frequency of playing video games.

Finally, we applied the one-sample Wilcoxon signed-rank test to determine whether the median for each ordinally evaluated statement differed significantly from neutral. We performed initial data processing with Microsoft Excel (Microsoft Corporation, Redmond, Washington, DC, USA). To generate the statistical report, we used R v4.0.5 (R Foundation for Statistical Computing, Vienna, Austria). A *p*-value < 0.05 was considered to indicate statistical significance. To create figures and graphs, we utilised GraphPad v9.3.1 (GraphPad Software Inc., San Diego, CA, USA).

We conducted a pilot study to determine the sample size, including six participants at the University Hospital Zurich. We used 1000 simulated data sets based on the pilot study’s results (rate ratio of 0.99 for Visual Blood compared to ABG printouts) and calculated mixed Poisson models with modality (Visual Blood or ABG printout) as a covariate with those data sets. The simulations showed that 50 participants were needed to show noninferiority with a noninferiority margin rate ratio of 0.9, a power of 95%, and a significance level of 0.05.

## 3. Results

**Figure 2:**
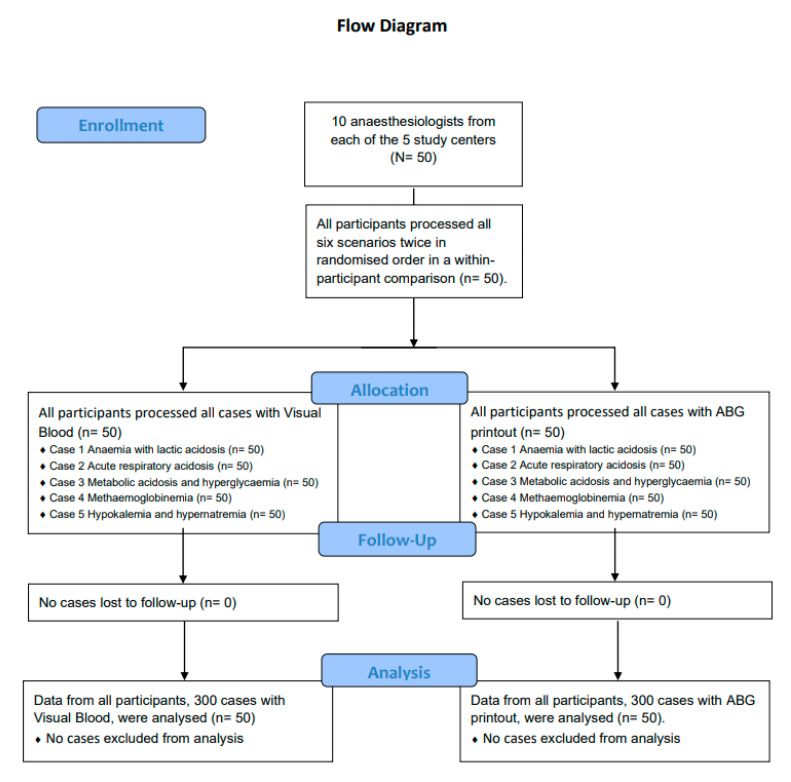
Flow diagram showing participant recruitment, allocation breakdown of scenarios with Visual Blood and scenarios with an ABG printout, follow-up, and data analysis. We modified the CONSORT 2010 flow diagram to fit the study design and the within-participant comparison of this study. ABG, arterial blood gas.

Between June and August 2021, we recruited a total of 50 participants drawn from the anaesthesia staff of the five study centres. All participants processed all six scenarios twice, once as Visual Blood and once as a conventional ABG printout, resulting in 600 assessed cases (Figure 2). The primary outcome dataset consisted of 5400 evaluated parameters per modality. The secondary outcomes of clinical diagnosis, diagnostic confidence, and perceived workload consisted of 300 ratings per modality. Table 1 displays additional study and participant characteristics.

### 3.1. Correct ABG Parameter Perception

Regarding the primary outcome, the participants correctly perceived 4008 of 5400 (74.2%) ABG parameters using Visual Blood, compared to 4177 of 5400 (77.4%) ABG parameters using ABG printouts.

**Figure 3:**
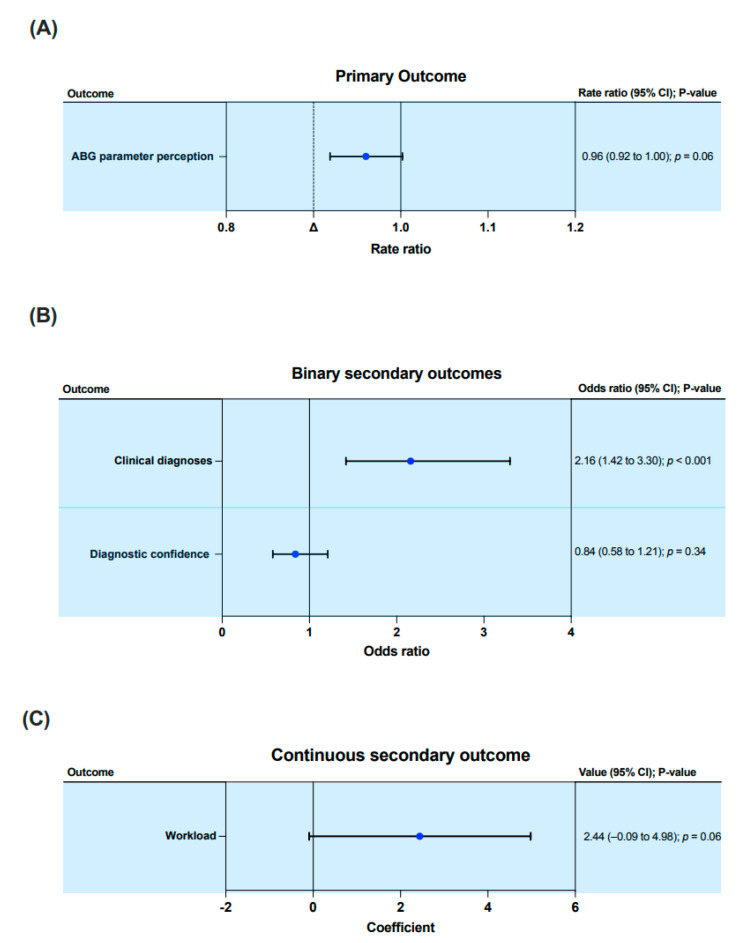
(**A**) Forest plot presenting the results of the mixed Poisson regression model for the primary outcome, ABG parameter perception. Δ shows the noninferiority margin at a rate ratio of 0.9. (**B**) Forest plots picturing the results of the mixed logistic regression models for the binary secondary outcomes: Clinical diagnosis and diagnostic confidence. (**C**) Coefficient for the outcome perceived workload, resulting from a linear mixed model, comparing Visual Blood and conventional printout. We measured the workload with the National Aeronautics and Space Administration Task Load Index (NASA TLX; from 0 to100). A positive coefficient means a higher workload using Visual Blood. CI, confidence interval.

The mixed Poisson regression model analysing the primary outcome, ABG parameter perception, yielded a rate ratio of 0.96 (95% CI, 0.92–1.00; *p* = 0.06) using Visual Blood compared to the ABG printout (Figure 3). The lower confidence interval of 0.92 was above the predefined noninferiority margin of 0.9, which showed noninferiority of Visual Blood for the primary outcome. Regarding the potentially relevant covariates, there was no evidence of an effect of age, gender, work experience, study centre, and self-rated frequency of playing video games on the primary outcome (all *p*-values > 0.05). However, in one scenario, the participants perceived significantly fewer ABG parameters than in the other five, independent of the used modality and the sequence of the scenarios (Table S1 in Supplementary Materials).

**Figure 4:**
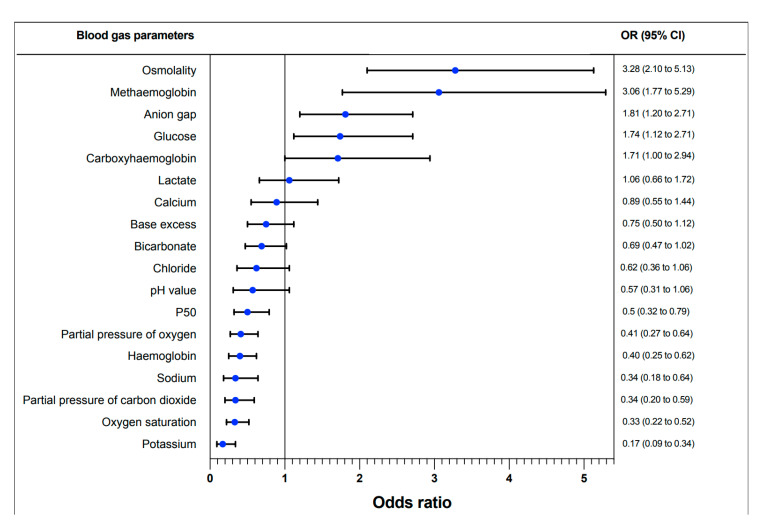
Graphical presentation of the crude matched odds ratios calculated for all blood gas parameters displayable with Visual Blood as a Forest plot. The odds ratios (OR) are presented as a dot with respective 95% confidence intervals (CI) shown as horizontal extensions. The y-axis specifies the particular parameter, while the x-axis offers the OR. As indicated by an odds ratio above one, Visual Blood seemed better in displaying Lactate, Carboxyhaemoglobin, Glucose, Anion gap, Methaemoglobin, and Osmolality.

Looking at the perception of each ABG parameter individually, the crude matched odds ratios showed that six of the eighteen parameters were better perceived with Visual Blood than with ABG printouts (Figure 4).

### 3.2. Correct Clinical Diagnoses

Regarding the secondary outcome correct clinical diagnosis, we found a higher number of correct diagnoses when using Visual Blood (254 of 300; 84.7%) than when participants worked with ABG printouts (219 of 300; 73.0%).

The mixed logistic regression model showed a strong difference between the two modalities that favoured Visual Blood (Figure 3). The odds of picking the correct clinical diagnosis were approximately two times higher when the participants used Visual Blood (OR, 2.16; 95% CI, 1.42–3.29; *p* < 0.001) compared to ABG printouts.

There was no evidence of an effect of age, gender, work experience, scenario, and self-rated frequency of playing video games on this outcome (all *p*-values > 0.05). However, there was evidence of performance differences between study centres concerning the outcome correct clinical diagnosis (Table S1 in Supplementary Materials).

### 3.3. Perceived Diagnostic Confidence

The mixed logistic regression models used to analyse diagnostic confidence showed no difference between the two modalities (OR, 0.84; 95% CI, 0.58–1.21; *p* = 0.34 and Figure 3).

### 3.4. Perceived Workload

The linear mixed model used to evaluate the perceived workload revealed weak evidence for Visual Blood causing a higher workload than ABG printouts (Coefficient, 2.44; 95% CI, −0.09–4.98; *p* = 0.06 and Figure 3).

### 3.5. Participant Opinions on Visual Blood and Virtual Reality in Clinical Practice

**Figure 5:**
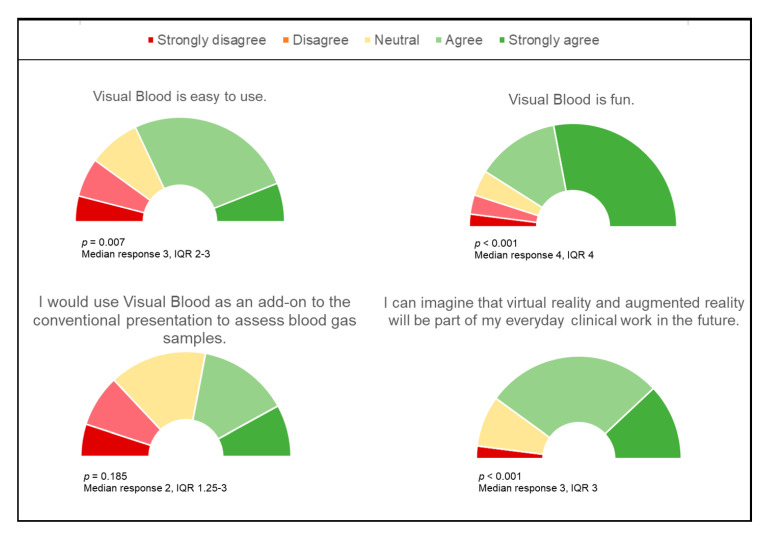
Half doughnut charts display the participants’ opinions concerning Visual Blood and virtual reality. The results are shown as the median and interquartile range (IQR). We used the Wilcoxon signed-rank test to determine whether the answers differed from neutral. Strongly disagree (1); Disagree (2); Neutral (3); Agree (4); Strongly agree (5). N = 50 in each statement.

A total of 42 of 50 (84%) participants agreed or strongly agreed that Visual Blood was fun to use, and 32 of 50 (64%) participants agreed or strongly agreed that Visual Blood was easy to use. A minority of 22 participants (44%) agreed or strongly agreed that they would use Visual Blood as an add-on to a conventional presentation of a blood gas sample. Concerning the statement if they could imagine that VR and augmented reality will be part of everyday clinical work in the future, 40 of 50 (80%) participants agreed or strongly agreed. Figure 5 illustrates the four statements that sampled the participants’ general opinions about Visual Blood and VR.

## 4. Discussion

This study compared the new three-dimensional VR visualisation technique, Visual Blood, to the conventional presentation of ABG results as printouts. Although participants did not perceive individual ABG parameters better, using Visual Blood resulted in more correct diagnoses than conventional ABG printouts.

Furthermore, there was no or, respectively, weak evidence for a difference in diagnostic confidence and perceived workload between the two modalities. Finally, the participants appraised Visual Blood as fun and easy to use and considered VR techniques to become part of the daily medical routine.

The participants rated fewer blood gas parameters correctly when using the VR presentation instead of the ABG printouts. One possible explanation is that unfamiliarity with the technical systems (VR headset and Visual Blood) reduced the participants’ concentration on the task, making memorising the individual blood gas parameters even more challenging. Accordingly, there is evidence that an unfamiliar environment or lack of practice may negatively affect performance [25,26]. In addition, known problems with VR headsets, including simulator sickness and insufficient wear comfort and fitting, may explain the result [19]. Although we used the spacer provided for the participants with prescribed glasses, this group sometimes had problems adjusting the VR headset to visualize everything in focus.

Nevertheless, the study found that 6 of the 18 parameters were better perceived with Visual Blood. It was noticeable here that methaemoglobin and carboxyhaemoglobin, two deviations rarely found in clinical practice, were better retrieved with Visual Blood, and there was more uncertainty when processing the ABG printouts. This might indicate that, especially in the case of a lack of familiarity, the perception of abnormalities is strengthened by Visual Blood. Identifying strengths in some parameter visualisations and corresponding weaknesses in others offers us valuable information for improving the design of specific Visual Blood components for a second prototype.

Visual Blood enabled the participants to find the correct clinical diagnosis with twice the odds of conventional ABG printouts. This is an important finding that supports the core competencies of the visualisation technique; these core competencies incorporate the basic ideas that a graphical data display and a system interface optimised according to user-centred design principles for the presentation of medical data support situation awareness and, eventually, decision-making [7,10,21]. Even though the participants could retrieve fewer individual parameters correctly, a clinical picture automatically assembled in their minds when using Visual Blood. As a result, the essential information for the clinical diagnosis was better transferred, allowing further detailed diagnostics or initiation of treatment.

Breaking down the concept of situation awareness described by Mica Endsley [6], which is composed of three levels, we identified room for improvement in several Visual Blood components concerning Level 1, “perception”. In contrast, in this study, the visualisation technique strengthened Level 2, comprehending the composition of diverse values. This finding is conclusive with the results of another visualisation technique invented for displaying patient monitoring data as a patient avatar, the Visual Patient [15]. A high-fidelity simulation study with Visual Patient found a higher probability of verbalising the cause of an emergency when using Visual Patient than when using a state-of-the-art patient monitor that contains numbers and waveforms [16]. We did not test Visual Bloods’ influence on situation awareness Level 3, which comprises the projection of future states and events such as treatment choices. The investigation of situation awareness Level 3 builds the ground for further studies.

Although VR applications are still rare in medical practice, one promising finding was that there was no or, respectively, little evidence for a difference between the new technique and ABG printouts in terms of perceived diagnostic confidence and perceived workload. One might have expected that a new, unfamiliar tool would lead to higher workload and less diagnostic confidence. Instead, these results suggest that the user-centred design principles incorporated into Visual Blood successfully transferred the information to the decision-makers quickly and with low cognitive demand [7]. Applying these principles when designing cognitive aids, visualisations, and sounds improve the performance of medical personnel by lowering workload and increasing diagnostic confidence [11,12,27,28,29].

Most participants in the study considered Visual Blood fun and easy to learn and, in general, envisioned VR and augmented reality as a part of everyday clinical practice in the future, although known technology problems were observed during the study. For example, currently used head-mounted displays usually weigh several hundred grams and produce plenty of heat. Therefore, long-term wear comfort is limited. In addition, for safety reasons, it is not possible to wear VR eyewear in the operating area at all times as the surroundings cannot then be observed. Furthermore, simulator sickness, which presents with nausea, headache, or vertigo, is a problem in some users. However, the rapid development of information technologies such as smartphones in the last two decades allows us to assume that improved VR and augmented reality hardware will be available soon that will smoothly integrate into everyday life and achieve maximum potential. Overall, qualitative studies have already yielded promising results in educating patients and physicians by considering a virtual environment as a helpful, encouraging, and joyful training medium. This encourages our attempt with Visual Blood to integrate these modern technologies into clinical practice.

### Strengths and Limitations

This study has several limitations. First, the data collection occurred in a protected, computer-based environment that excluded clinical surroundings. In this artificially created situation, the clinical presentation and the patient’s medical history were missing as were the real-life healthcare challenges that weighed on the participants. However, the standardisation of the simulated ambience enabled us to focus entirely on investigating the new technique and reduced unknown influencing variables. In addition, simulation studies are considered an optimal environment to train and assess new methods [30,31]. Second, we performed this study in tertiary care hospitals in Europe. Therefore, the results may differ elsewhere in the world. However, we consider this unlikely, as interpreting ABG results is common for every physician worldwide, and ABG testing equipment is widely available. Generally, it is difficult to ensure equal difficulty for all scenarios, since the participants’ individual interests and knowledge levels differ. Due to their alignment with clinical diagnoses, the quantity of abnormal parameters varies between the scenarios. Our statistical analysis confirmed that the particular scenario affected performance, confidence, and NASA TLX. However, the fact that the participants processed all scenarios in both modalities in individual sequential randomisation mitigates this limitation. Finally, we underestimated the known limitations of VR and augmented reality visualisations in the study design [19]. A study using VR or augmented reality headsets should assess the incidence of simulator sickness and exclude participants with prescription glasses or ask them to wear contact lenses if possible.

An apparent strength of the study was the multicentre design across three countries and the broad participant spectrum in terms of age and work experience, minimising selection bias. Further, we adapted the printouts’ layout precisely to the conditions of the respective study centre to guarantee a high degree of familiarity. Furthermore, we used a modern state-of-the-art VR headset that works without additional sensors and computers. With a few hundred grams of weight and a comfortable headband, even longer VR sessions can be spent comfortably. Nevertheless, we must admit that we could not prevent known limitations of the technique.

## 5. Conclusions

This multicentre, multinational study compared the innovative Visual Blood to the conventional ABG printout in a computer-based simulation trial. Although the participants did not perceive the individual ABG parameters better, the use of Visual Blood resulted in more correct diagnoses than the use of the conventional ABG printouts. This finding suggests that the user-centred display in Visual Blood allows for a higher level of situation awareness that goes beyond the mere perception of individual parameters. Although ABG is a global and frequently used tool to evaluate a patient’s status quickly, research has neglected the design aspects of the result presentation. We believe it is time to make a change and evolve towards Visual Blood. The overall goal is to create a user-friendly ABG display that allows for better situation awareness, better decision-making, and, thus, optimised patient care.

## Figures and Tables

**Table 1:** Study and participant characteristics. IQR, Interquartile range; ABG, Arterial blood gas.

Study centres, n	5
Participants per study centre, n	10
Age in years, median (IQR 25/75, (range))	31.0 (28/40.8 (25–56))
Work experience in years, median (IQR 25/75, (range))	5.0 (2/10 (1–30))
Gender female, n (%)	31 (62%)
Resident physician, n (%)	28 (56%)
Self-rated theoretical ABG skills (0 = novice, 100 = expert), median (IQR 25/75, (range))	70.5 (60/83 (31–100))
Self-rated frequency of playing video games (0 = never, 100 = very often), median (IQR 25/75, (range))	6.0 (0/30.8 (0–87))

## Data Availability

The data are available from the corresponding author.

## Supplementary Materials

The following supporting information can be downloaded at: https://www.mdpi.com/article/10.3390/bioengineering10030340/s1; Video S1: “Visual Blood Educational Experience” video explaining Visual Blood. Table S1: Overview of all scenarios with the respective deranged and normal parameters, and results for mixed Poisson and logistic regression models. Figure S1: Different presentations of acute respiratory acidosis.
