# Supplementary material for: Visual Blood, Visualisation of Blood Gas Analysis in Virtual Reality, Leads to More Correct Diagnoses: A Computer-Based, Multicentre, Simulation Study
Source: Bioengineering (Basel). 2023 Mar 8;10(3):340. doi: 10.3390/bioengineering10030340 (PMC10044755; doi:10.3390/bioengineering10030340)
Supplement: Supplementary file 1 [file bioengineering-10-00340-s001.zip › Table S1.pdf]

**Table S1.** Overview of all scenarios with the respective deranged and normal parameters. Orange: too low; green: safe; purple: too high.

| **Scenario** | **1** | **2** | **3** | **4** | **5** | **6** |
|---|---|---|---|---|---|---|
| **Clinical diagnosis** | Anaemia with lactic acidosis | Acute respiratory acidosis | Metabolic acidosis in hyper-glycaemia | Methaemo-globinemia | Hypo-kalaemia and hyper-natriaemia | Hypo-xaemia |
| **Value** | | | | | | |
| pH | | | | | | |
| pCO2 | | | | | | |
| pO2 | | | | | | |
| P50 | | | | | | |
| sO2 | | | | | | |
| Haemoglobin | | | | | | |
| COHb | | | | | | |
| MetHb | | | | | | |
| Potassium | | | | | | |
| Sodium | | | | | | |
| Chloride | | | | | | |
| Calcium | | | | | | |
| Anion gap | | | | | | |
| Lactate | | | | | | |
| Glucose | | | | | | |
| Osmolality | | | | | | |
| Base excess | | | | | | |
| Bicarbonate | | | | | | |

*Abbrevations: pCO2: partial pressure of carbon dioxide; pO2: partial pressure of oxygen; sO2: oxygen saturation; COHb: carboxyhaemoglobin; MetHb: methaemoglobin.*
