# Supplementary figures and images for: Visual Blood, Visualisation of Blood Gas Analysis in Virtual Reality, Leads to More Correct Diagnoses: A Computer-Based, Multicentre, Simulation Study
Source: Bioengineering (Basel). 2023 Mar 8;10(3):340. doi: 10.3390/bioengineering10030340 (PMC10044755; doi:10.3390/bioengineering10030340)

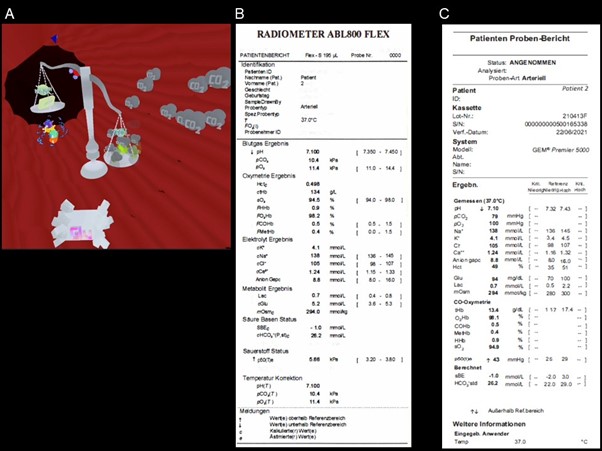

Supplement: Supplementary file 1 [file bioengineering-10-00340-s001.zip › Figure S1.jpg]
